# A Retrospective Study of Cefiderocol Utilization and Associated Outcomes at an Academic Medical Center

**DOI:** 10.3390/idr17050112

**Published:** 2025-09-11

**Authors:** Samantha G. Rauch, Michelle H. Potter, Emir Kobic

**Affiliations:** Department of Pharmacy, Banner University Medical Center Phoenix, Phoenix, AZ 85006, USA; samantha.rauch@bannerhealth.com (S.G.R.);

**Keywords:** multidrug-resistant organism, cefiderocol, patient outcomes

## Abstract

Background: This study aimed to describe cefiderocol prescription and associated patient outcomes at a U.S. academic medical center for various multidrug-resistant organisms (MDRO). Notably, limited real-world data exist on cefiderocol’s clinical use in regions where metallo-β-lactamase (MBL)-producing organisms are prevalent. Methods: A retrospective chart review was conducted on adult inpatients who received ≥24 h of cefiderocol between January 2023 and July 2024. Data collected included microbiology, carbapenemase type (CARBA-5), treatment indication, susceptibility profiles, and clinical outcomes: 30-day mortality, re-infection, and re-admission. Descriptive statistics were used. Results: Seventy-six patients were included, with most receiving cefiderocol for carbapenem-resistant Enterobacterales (CRE) (63%) or *P. aeruginosa* (17%) infections. Overall, 96% of cases met institutional prescribing criteria. NDM was the predominant carbapenemase (77% of CRE isolates). Cefiderocol was used definitively in 68% of cases. The median duration of therapy was 7 days. Thirty-day mortality was 20%, highest among patients with *A. baumannii* complex (33%). Re-infection and re-admission occurred in 21% and 32% of patients, respectively. Susceptibility to cefiderocol was highest for *P. aeruginosa* (100%), *Stenotrophomonas* (100%), and CRE (88%), but only 50% for *A. baumannii* complex. Conclusions: Cefiderocol was primarily used in accordance with institutional criteria and demonstrated favorable susceptibility against most target pathogens. However, poor outcomes in *A. baumannii* complex infections highlight the need for cautious use and the need for rapid diagnostics for early targeted therapy in multidrug-resistant infections.

## 1. Introduction

The World Health Organization (WHO) classifies priority pathogens as critical, high, or medium to promote research and development (R&D) of new antibiotics [1]. The critical group includes *Acinetobacter baumannii* complex, *Pseudomonas aeruginosa*, and various Enterobacterales (including *Klebsiella pneumoniae* and *Escherichia coli*) as these organisms can often become resistant to many antibiotics, including carbapenems. *Stenotrophomonas maltophilia*, a bacteria intrinsically resistant to carbapenems with high propensity to become a multidrug-resistant organism (MDRO), is noted as an emerging pathogen due to rising incidence and limited R&D of new therapeutics. Over the past decade, cefiderocol has been the only novel FDA-approved β-lactam that has demonstrated potent in vitro activity against all of these pathogens, even when carbapenem resistance is detected [2].

Cefiderocol is a cephalosporin antibiotic with FDA-approved indications for complicated urinary tract infection (cUTI), pyelonephritis, hospital-acquired bacterial pneumonia (HABP), or ventilator-associated bacterial pneumonia (VABP) caused by a susceptible Gram-negative organism [3]. In the APEKS-cUTI and APEKS-NP registrational trials, cefiderocol demonstrated clinical efficacy against Enterobacterales species, *Pseudomonas aeruginosa*, and *Acinetobacter baumanni* complex, leading to its FDA approval for the treatment of these organisms [3]. While the small number of included patients with carbapenem-resistant Enterobacterales (CRE) infections achieved clinical efficacy in the APEKS-NP trial, efficacy concerns with cefiderocol use in carbapenem-resistant organism (CRO) infections have been reported. The CREDIBLE-CR trial was a pathogen-directed randomized controlled trial with CRO infections that reported higher rates of all-cause mortality in patients receiving cefiderocol for the treatment of carbapenem-resistant *Acinetobacter baumannii* complex (CRAB) infections when compared to patients receiving best available therapy (BAT) [4]. However, no significant differences in all-cause mortality were seen between patients receiving cefiderocol for the treatment of monomicrobial CRE or *Pseudomonas* infections when compared to patients receiving BAT, which was most often a colistin backbone regimen. A warning of increase in all-cause mortality in patients with CRO infections is currently listed in the package insert, emphasizing the importance of the interim PROVE study, an observational retrospective multicenter real-world evaluation of cefiderocol use across 15 US sites, to complement data from registrational trials given the limited number of novel therapies available to treat these challenging infections [5]. Our single-center real-world use study supplements data from multicenter sites across the US, while also adding outcomes for a concerning regional trend in which New Delhi metallo-β-lactamase (NDM) has emerged as the predominant carbapenemase among CRE infections [6].

Our study is a descriptive analysis evaluating cefiderocol-prescribing practices at a single academic medical center, focusing on adherence to institutional guidelines as well as patient outcomes, including in-hospital 30-day mortality, 30-day microbiologic recurrence, and 30-day re-admission.

## 2. Materials and Methods

A retrospective chart review was conducted at a 777-bed academic medical center, evaluating inpatients treated with at least 24 h of cefiderocol between 1 January 2023 and 31 July 2024. The exclusion criteria were pregnancy, therapy < 24 h, and concomitant enrollment in an ongoing continuous renal replacement therapy cefiderocol study. Facility guideline criteria for cefiderocol use include strong suspicion of or previously documented infection due to difficult-to-treat-resistant (DTR) *Pseudomonas*, CRAB, or metallo-β-lactamase-producing CRE in the previous 12 months, or active infections due to DTR *Pseudomonas*, CRAB, metallo-β-lactamase-producing CRE, or MDR *Stenotrophomonas* with susceptibilities to cefiderocol ordered or confirmed.

Baseline characteristic and outcome data were collected from the centralized electronic health record. Outcomes of interest included 30-day mortality from cefiderocol initation, 30-day infection recurrence from therapy completion, and 30-day re-admission from therapy completion. Prior MDRO infection was defined as DTR *Pseudomonas*, CRE, CRAB, *S. maltophilia*, and/or any extended-spectrum β-lactamase (ESBL)-producing organisms within the 12 months prior to cefiderocol initiation.

Antimicrobial susceptibilities were determined by automated susceptibility testing (Vitek or Microscan) using interpretive criteria from Clinical Laboratory Standards Institute (CLSI) M100-ED35:2025, while cefiderocol testing was performed with Kirby–Bauer disk diffusion using FDA zone diameter breakpoints for Enterobacterales, *Acinetobacter baumanni* complex, and *Pseudomonas aeruginosa*; for *Stenotrophomonas maltophilia,* CLSI zone diameters were used. Carbapenemase screening was performed using the CARBA-5 assay, which was run whenever an Enterobacterales is non-susceptible to any carbapenem and is a rapid phenotypic diagnostic test that detects the presence of KPC, NDM, VIM, IMP, and OXA-48; this screening was implemented at our facility in November 2023 and results were therefore only available for patients admitted after this time period.

Statistical analysis included descriptive statistics only. Medians with interquartile ranges were reported for continuous variables and counts with percentages were reported for categorical variables. During manuscript preparation, ChatGPT-5 (OpenAI, San Francisco, CA, USA) was used in a limited capacity to suggest alternative phrasings and improve readability of certain sections. The tool was not used to create data, perform analyses, or generate original scientific content. All suggestions from the AI were critically assessed, revised as needed, and finalized by the authors.

## 3. Results

**Figure 1:**
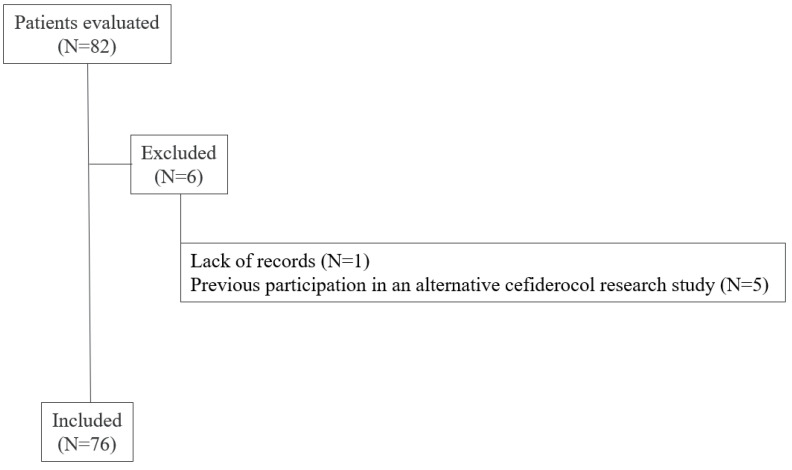
Flowchart of inclusion and exclusion criteria.

A total of 82 patients were reviewed for inclusion, and 6 were excluded, 5 of them due to previous participation in an alternative research study and 1 due to lack of records from outside hospital prior to transfer. A total of 76 patients met inclusion criteria and were analyzed (Figure 1).

Baseline characteristics are shown in Table 1. The median age was 60 years old. Patients were primarily male, Caucasian, and non-Hispanic or Latino. The median Charlson Comorbidity Index (CCI) was 10 and the median SOFA score at baseline was 5. Overall, 33 patients (43%) were admitted to the ICU including 8 patients (62%) with DTR *Pseudomonas*, 18 patients (38%) with CRE, 6 patients (67%) with CRAB, and 2 patients (66%) with *S. maltophilia*. The most common indications included UTI, pneumonia, and bacteremia. Patients with DTR *Pseudomonas* had more respiratory infections compared to CRE and CRAB. The most common indication for CRE was UTI. Most patients did not have a documented MDRO infection within the previous 12 months.

The most common organism was *K. pneumoniae*, most frequently NDM positive (70%) (see Table 2). All tested isolates of DTR *Pseudomonas* and *Stenotrophomonas* were cefiderocol-susceptible, whereas only 88% of tested CRE isolates and 50% of tested CRAB isolates were cefiderocol-susceptible. Cefiderocol remained the agent with the highest susceptibility amongst the novel β-lactams (see Table 3).

Results and outcomes are shown in Table 4. Almost all patients (99%) received an infectious diseases (ID) consult during their admission. Cefiderocol was used as definitive treatment in 68%. Of the 24 patients (32%) receiving cefiderocol as empiric treatment, 7 (29%) did not ultimately grow an MDRO.

Institutional restriction criteria for use of cefiderocol were met in most patients (96%). In total, 22 patients (29%) were switched to alternative agents during their admission. De-escalation of therapy occurred in 14 (64%) of these patients due to lack of MDRO identification on cultures while 8 patients (36%) were switched to alternative MDRO therapies including ceftazidime-avibactam/aztreonam, meropenem-vaborbactam, ceftolozane-avibactam, eravacycline, and tigecycline.

Median time to initiation of cefiderocol was 3 days. The median length of stay was 19 days overall. DTR *Pseudomonas* had the longest median length of stay (47 days) and duration of therapy (12 days) compared to other groups. Mortality within 30 days of cefiderocol initiation occurred in 15 patients (20%), with CRAB showing the highest rates of mortality. In total, 16 patients (21%) experienced infection recurrence within 30 days of therapy completion with the highest re-infection rates occurring in patients with DTR *Pseudomonas* infections. A total of 24 patients (32%) were re-admitted within 30 days of therapy. DTR *Pseudomonas*, CRAB, and *Stenotrophomonas* had the highest rates of re-admission with half or more of surviving patients re-admitted within 30 days.

## 4. Discussion

This analysis represents a real-world evaluation of cefiderocol prescribing at a large, single-center U.S. academic medical center. In 66% of cases, prescribing followed FDA-approved indications for urinary tract infection (UTI) and pneumonia, while the remaining were for off-label indications such as bacteremia, SSTI, and intra-abdominal infections. The majority of patients had CROs isolated, most commonly *Klebsiella pneumoniae*, with NDM identified as the predominant carbapenemase. Although overall cefiderocol susceptibility was high (88%) and UTI was the most frequent indication, the 30-day mortality rate in our cohort was 19%, compared to 12% 28-day mortality observed in the cUTI subgroup of the CREDIBLE-CR trial, where carbapenem-resistant *K. pneumoniae* was also the most common pathogen [4]. While SOFA scores were comparable between studies, differences in clinical outcomes may be attributed to delays in initiating appropriate therapy. In addition, NDM-5 among *K. pneumoniae* has been detected in our region and is associated with reduced cefiderocol susceptibility, although our study was not powered to detect outcome differences based on susceptibilities [7].

Most patients received cefiderocol as definitive therapy, suggesting culture and susceptibility results prompted therapy initiation. Empiric prescribing accounted for 32% of cases, a proportion consistent with the 42% of patients who had a documented MDRO within the prior year. The high rates of patients with MDRO history indicate that though empiric prescribing was common, it was overall appropriate, which is also in line with the 96% of patients overall who met institutional restriction criteria. In comparison, interim analyses from the PROVE study, a post-marketing real-world observational study, reported similar prevalence of prior CRO or colonization (32.4%), but a lower rate of empiric prescribing (11.1%) [5]. In comparison, our cohort had higher severity of illness (median CCI of 10 vs. 2). Another important distinction, the median time to cefiderocol initiation in PROVE was 5 days, slightly longer than the 3 days observed in our study. Despite these differences, 30-day in-hospital mortality rates were similar between the studies (20% vs. 18.4%) whereas the CREDIBLE-CR trial saw slightly higher 28-day all-cause mortality rates in patients treated with cefiderocol versus BAT, a colistin backbone regimen (24.8% vs. 18.4%) [4,5].

Our study confirmed notably lower susceptibility among CRAB isolates (50%), with a corresponding 30-day mortality rate of 33% in this group. This is lower than the 28-day all-cause mortality of 50% reported in the cefiderocol arm of the CREDIBLE-CR trial for CRAB infections, where majority of patients had severe presentations such as pneumonia or bacteremia [4]. In contrast, four of our nine patients with CRAB had skin/soft tissue or osteomyelitis infections, which generally carry a lower risk of 28-day mortality, potentially contributing to the observed difference. In the colistin-based arm of CREDIBLE-CR, the mortality rate for CRAB was 19%; although no definitive cause for the difference was identified, the cefiderocol group had more patients over 65 years, more ICU admissions, and more cases of septic shock, which may have influenced outcomes [4]. Historically, mortality rates for CRAB infections treated with colistin-based regimens have typically ranged from 42 to 52%, while the ATTACK trial recently reported a 28-day mortality of 19% with sulbactam–durlobactam and 32% with colistin for nosocomial pneumonia, with both arms receiving imipenem for combination therapy [8]. Our findings highlight that outcomes in CRAB infections remain poor, and although our mortality rate was lower than CREDIBLE-CR, it reinforces ongoing concerns about the effectiveness of cefiderocol for CRAB infections. Accordingly, we believe cefiderocol should be used with caution in this setting and reserved for refractory cases, ideally in combination with other active agents when available. The median SOFA score among our CRAB patients was 5, comparable to the median of 6 in the CREDIBLE-CR cohort, suggesting similar illness severity. Median time to cefiderocol initiation was also longest in CRAB patients, which is a key contributor to poor outcomes. The combination of diagnostic lag and a critically ill population likely contributed to higher 30-day mortality and re-admission.

Patients with DTR *Pseudomonas aeruginosa* had the longest lengths of stay and longer therapy durations compared to other subgroups, reflecting greater clinical complexity. Ceftolozane-tazobactam remains the preferred agent for DTR *Pseudomonas aeruginosa* per institutional criteria, and cefiderocol is often reserved for cases with treatment failure or resistance. Consequently, longer hospitalizations in this group likely stem from delayed initiation following failure of first-line therapy with ceftolozane-tazobactam, and recurrent infections due to prolonged antimicrobial exposure. The median duration of cefiderocol therapy across all patients was 7 days, with a median of 12 days for those with DTR *Pseudomonas*, consistent with real-world practice for this organism in critically ill patients. These findings reflect appropriate prescribing patterns based on infection type and severity. They also provide real-world prescribing behaviors and outcomes for patients who often fall in the exclusion criteria of previous cefiderocol registrational trials with patients whose infection began >72 h prior to cefiderocol start [4,9,10]. Prior studies have shown that delays in appropriate antimicrobial therapy of >48 h are associated with worse outcomes, including increased length of stay, mortality, and cost [11,12]. The delay in initiation observed not only in the PROVE study but also our own may limit the drug’s clinical efficacy in real-world use.

Although based on a small sample, all *S. maltophilia* isolates in our study were susceptible to cefiderocol, consistent with its potent in vitro surveillance data [2]. In a preclinical neutropenic rabbit model of *S. maltophilia* pneumonia, cefiderocol demonstrated superior bacterial eradication and survival rates compared to trimethoprim-sulfamethoxazole, with 87% survival in the cefiderocol group versus 25% with TMP-SMX and 0% in untreated controls [13]. The Infectious Diseases Society of America (IDSA) suggests using two active agents for *S. maltophilia* and includes cefiderocol as a potential option despite limited clinical data [14]. In the CREDIBLE-CR trial, four of five patients with *S. maltophilia* experienced 28-day mortality, with three cases involving polymicrobial infections that included *Acinetobacter baumannii* complex [5]. A multicenter real-world study of cefiderocol treatment from France, which included the largest cohort of immunocompromised patients with *S. maltophilia* infections treated with cefiderocol to date, reported a 28-day mortality rate of 37.7% and frequent relapses. Notably, cefiderocol resistance developed in one *S. maltophilia* case, with two additional cases showing resistance by day 90 [15]. Similarly, a subgroup analysis from the PERSEUS study in Spain observed a 28-day all-cause mortality rate of 30% [16]. Our study included only three *S. maltophilia* patients, with one (33%) experiencing 30-day mortality. Two of the three patients grew *S. maltophilia* as a component of polymicrobial infections; as *S. maltophilia* was not thought to be the primary pathogen, these two patients did not receive combination therapy as is suggested in the IDSA guidelines. However, one patient had persistent *S. maltophilia* growth on first respiratory and then blood cultures despite trimethoprim/sulfamethoxazole therapy—cefiderocol was added on to trimethoprim/sulfamethoxazole in this patient, but they ultimately expired. There is currently a need for robust clinical evidence for cefiderocol treatment for *S. maltophilia*.

A major limitation of our study is the retrospective observational design as it may introduce bias. Our study is also limited by the potential of unidentified risk factors that may have impacted mortality, re-infection, and re-admission rates. Additionally, our small sample size especially for CRAB, DTR *Pseudomonas*, and *Stenotrophomonas* limits the generalizability of our data. It is important to mention our facility, as of November 2023, implemented routine CARBA-5 testing which alerts providers of the specific cabapenemases present prior to sensitivity panel results. Therefore, carbapenemase data was not available for our entire CRE cohort, potentially delaying time to initiation of therapy in the earlier patients.

## 5. Conclusions

The bulk of cefiderocol prescribing at our institution was for CROs and 96% met institutional criteria of use. The majority of prescribing was found to be definitive, guided by culture and susceptibility results. These findings provide additional real-world evidence on patient outcomes in a cohort predominantly affected by NDM-producing organisms, a population that was underrepresented in prior trials. Despite this difference, the overall results were consistent with those observed in the interim PROVE study and the CREDIBLE-CR trial. For daily practice, our data highlight the longest delays in therapy initiation and highest mortality rates occurred in patients with CRAB and Stenotrophomonas infections. Although the sample size was small, it signals the need for continued caution when prescribing cefiderocol in this setting. These results reinforce the importance of incorporating rapid diagnostic testing and timely escalation or de-escalation into clinical workflows to optimize outcomes in patients with multidrug-resistant infections.

## Figures and Tables

**Table 1:** Patient demographics.

	All Patients (*n* = 76) *	DTR *Pseudomonas* (*n* = 13)	CRE (*n* = 48)	CRAB (*n* = 9)	*Stenotrophomonas* (*n* = 3)
Age (years) Median (IQR)	60 (20)	50 (28)	62 (19)	52 (23)	48 (6)
**Sex**					
Male	47 (62%)	9 (69%)	30 (63%)	3 (33%)	1 (33%)
**Race**					
Caucasian	57 (75%)	9 (69%)	36 (75%)	9 (100%)	3 (100%)
Black or African American	16 (21%)	4 (31%)	10 (21%)	--	--
≥2 Races	3 (4%)	--	2 (4%)	--	--
**Ethnicity**					
Hispanic or Latino	17 (22%)	5 (38%)	9 (19%)	2 (22%)	--
Non-Hispanic or Latino	59 (78%)	8 (62%)	39 (81%)	7 (78%)	3 (100%)
CCI	10 (5)	11 (4)	10 (5)	9 (2.5)	8 (10)
SOFA	5 (6)	7 (4)	4.5 (4)	5 (4)	10 (6)
**Unit**					
ICU	33 (43%)	8 (62%)	18 (38%)	6 (67%)	2 (66%)
Non-ICU	43 (57%)	5 (38%)	30 (63%)	3 (33%)	1 (33%)
**Site of Infection º**					
UTI	27 (36%)	4 (31%)	22 (46%)	--	
Bacteremia	20 (26%)	2 (15%)	16 (33%)	1 (11%)	1 (33%)
SSTI	7 (9%)	--	3 (6%)	3 (33%)	1 (33%)
Intra-abdominal	5 (7%)	--	2 (4%)	1 (11%)	
Osteomyelitis	2 (3%)	--	--	1 (11%)	
CNS	1 (1%)	--	1 (2%)	--	
Respiratory	23 (30%)	9 (69%)	9 (19%)	3 (33%)	2 (66%)
Other	2 (3%)	--	2 (4%)	--	
**Prior MDRO ˆ**					
DTR *Pseudomonas*	11 (14%)	5 (38%)	3 (6%)	--	
CRE	19 (25%)	4 (31%)	12 (25%)	3 (33%)	1 (33%)
CRAB	7 (9%)	3 (23%)	4 (8%)	2 (22%)	
ESBL	3 (4%)	5 (38%)	3 (6%)	2 (22%)	1 (33%)
None	47 (62%)	5 (38%)	31 (65%)	4 (44%)	

CCI, Charlson Comorbidity Index; CNS, central nervous system; CRAB, carbapenem-resistant *Acinetobacter baumannii* complex; CRE, carbapenem-resistant Enterobacterales; DTR, difficult-to-treat-resistant; ESBL, extended-spectrum β-lactamase; ICU, intensive care unit; IQR, interquartile range; MDRO, multidrug-resistant organism; SOFA, Sequential Organ Failure Assessment; SSTI, skin and soft tissue infection; UTI, urinary tract infection. * Patients not included in the rest of the table include those with non-MDROs or no growth on cultures; º Some patients had more than one site of infection; ˆ Some patients had multiple prior MDRO infections.

**Table 2:** Microbiology results.

CARBA-5 Result (*n* = 39)	
KPC	1 (3%)
NDM	30 (77%)
VIM	1 (3%)
Negative	7 (18%)
Organisms (*n* = 76) *	
*Pseudomonas* species	13 (17%)
*Klebsiella pneumoniae*	53 (70%)
*Enterobacter cloacae*	1 (1%)
*Escherichia coli*	2 (3%)
*Serratia marcescens*	3 (4%)
*Acinetobacter baumannii* complex	9 (12%)
*Stenotrophomonas maltophilia*	3 (4%)
Other	4 (5%)

* Some patients grew multiple organisms.

**Table 3:** Susceptibilities from clinical strains.

	Number of Strains	Cefiderocol	Meropenem	Imipenem	Ceftolozane/ Tazobactam	Ceftazidime/ Avibactam	Meropenem/ Vaborbactam
DTR *Pseudomonas*	13 *	8/8 (100%)	1/11 (9%)	1/7 (14%)	3/7 (43%)	3/9 (33%)	-
CRE	48 *	42/48 (88%)	0/47 (0%)	0/30 (0%)	N/T	1/18 (6%)	1/17 (6%)
CRAB	9 *	4/8 (50%)	0/8 (0%)	0/8 (0%)	-	-	-
Stenotrophomonas †	3	3 (100%)	-	-	-	-	-

- = No established CLSI breakpoint; N/T = not tested; * not all strains were tested for susceptibilities to all agents, denominators are included to denote how many were tested; † two of three strains were part of polymicrobial infections. All isolates were susceptible to trimethoprim/sulfamethoxazole and minocycline. Levofloxcin was resistant for one isolate and susceptible for the other two.

**Table 4:** Outcomes.

	All Patients (*n* = 76) *	DTR *Pseudomonas* (*n* = 13)	CRE (*n* = 48)	CRAB (*n* = 9)	*Stenotrophomonas* (*n* = 3)
ID Consulted	75 (99%)	12 (92%)	48 (100%)	9 (100%)	3 (100%)
Empiric vs. Definitive					
Empiric	24 (32%)	8 (62%)	9 (19%)	3 (33%)	--
Definitive	52 (68%)	5 (38%)	39 (81%)	6 (67%)	3 (100%)
Time to Initiation (days) Median (IQR)	3 (2)	3 (3)	3 (2)	4 (2)	5 (4)
Restriction Requirements Met	73 (96%)	12 (92%)	46 (96%)	9 (100%)	2 (66%)
Switched to Alternative Agent	22 (29%)	3 (23%)	8 (17%)	3 (33%)	1 (33%)
Duration of Therapy (days) Median (IQR)	7 (9)	12 (8)	7 (10)	6 (6)	10 (5)
Length of Stay (days) Median (IQR)	19 (29)	47 (49)	18 (17)	18 (24)	25 (30.5)
30-Day Mortality	15 (20%)	2 (15%)	9 (19%)	3 (33%)	1 (33%)
30-Day Re-Infection	16 (21%)	4 (36%)	10 (25%)	--	--
30-Day Re-Admission	24 (32%)	5 (38%)	15 (31%)	3 (33%)	2 (66%)

CRAB, carbapenem-resistant *Acinetobacter baumannii* complex; CRE, carbapenem-resistant Enterobacterales; DTR, difficult-to-treat-resistant; ID, infectious disease; IQR, interquartile range. * Patients not included in the rest of the table include those with non-MDROs or no growth on cultures.

## Abbreviations

The following abbreviations are used in this manuscript:

BAT	Best available therapy
CARBA-5	Carbapenemase screening assay that detects KPC, NDM, VIM, IMP, and OXA-48
CCI	Charlson Comorbidity Index
CLSI	Clinical Laboratory Standards Institute
CNS	Central nervous system
CRAB	Carbapenem-resistant *Acinetobacter baumannii* complex
CRE	Carbapenem-resistant Enterobacterales
CRO	Carbapenem-resistant organism
cUTI	Complicated urinary tract infection
DTR	Difficult-to-treat-resistant
ESBL	Extended-spectrum β-lactamase
FDA	U.S. Food and Drug Administration
HABP	Hospital-acquired bacterial pneumonia
ICU	Intensive care unit
ID	Infectious diseases
IQR	Interquartile range
MBL	Metallo-β-lactamase
MDR	Multidrug-resistant
MDRO	Multidrug-resistant organism
NDM	New Delhi metallo-β-lactamase
R&D	Research & Development
SOFA	Sequential Organ Failure Assessment
SSTI	Skin and soft tissue infection
UTI	Urinary tract infection
VABP	Ventilator-associated bacterial pneumonia
WHO	World Health Organization
